# Pleiotropic Roles of Atrial Natriuretic Peptide in Anti-Inflammation and Anti-Cancer Activity

**DOI:** 10.3390/cancers14163981

**Published:** 2022-08-17

**Authors:** Huafeng Fu, Jian Zhang, Qinbo Cai, Yulong He, Dongjie Yang

**Affiliations:** 1 Center for Gastrointestinal Surgery, The First Affiliated Hospital, Sun Yat-sen University, Guangzhou 510080, China; 2 Digestive Medicine Center, The Seventh Affiliated Hospital, Sun Yat-sen University, Shenzhen 518107, China; 3 Guangdong Provincial Key Laboratory of Digestive Cancer Research, Shenzhen 518107, China

**Keywords:** atrial natriuretic peptide, inflammation, anti-cancer agent, heart hormone, polypeptide

## Abstract

**Simple Summary:**

The relationship between inflammation and carcinogenesis, as well as the response to anti-tumor therapy, is intimate. Atrial natriuretic peptides (ANPs) play a pivotal role in the homeostatic control of blood pressure, electrolytes, and water balance. In addition, ANPs exert immune-modulatory effects in the tissue microenvironment, thus exhibiting a fascinating ability to prevent inflammation-related tumorigenesis and cancer recurrence. In cancers, ANPs show anti-proliferative effects through several molecular pathways. Furthermore, ANPs attenuate the side effects of cancer therapy. Therefore, ANPs have potential therapeutic value in tumors. Here, we summarized the roles of ANPs in diverse aspects of the immune system and the molecular mechanisms underlying the anti-cancer effects of ANPs, contributing to the development of ANP-based anti-cancer agents.

**Abstract:**

The atrial natriuretic peptide (ANP), a cardiovascular hormone, plays a pivotal role in the homeostatic control of blood pressure, electrolytes, and water balance and is approved to treat congestive heart failure. In addition, there is a growing realization that ANPs might be related to immune response and tumor growth. The anti-inflammatory and immune-modulatory effects of ANPs in the tissue microenvironment are mediated through autocrine or paracrine mechanisms, which further suppress tumorigenesis. In cancers, ANPs show anti-proliferative effects through several molecular pathways. Furthermore, ANPs attenuate the side effects of cancer therapy. Therefore, ANPs act on several hallmarks of cancer, such as inflammation, angiogenesis, sustained tumor growth, and metastasis. In this review, we summarized the contributions of ANPs in diverse aspects of the immune system and the molecular mechanisms underlying the anti-cancer effects of ANPs.

## 1. Introduction

**Figure 1:**
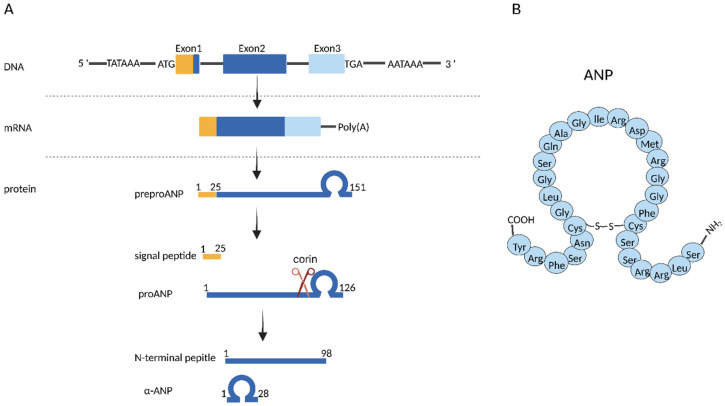
Schematic representation of human atrial natriuretic peptide gene structure and biosynthetic process. (**A**) biosynthetic process of ANPs. (**B**) primary structure of ANPs.

Natriuretic peptides (NPs) are a polypeptide hormone family that can be subdivided into four types: atrial natriuretic peptides (ANPs), brain natriuretic peptides (BNPs), type-C natriuretic peptides (CNPs), and dendroaspis natriuretic peptides (DNPs) [1,2]. The ANPs and BNPs are mainly synthesized in atrial and ventricular myocytes, whereas the CNPs are from endothelia cells and male genital glands [3]. DNPs were firstly identified in the venom of Dendroaspis angusticeps, or the green Mamba snake, and have similar structure and biological effects to ANPs, BNPs, and CNPs [2]. The human ANP preprohormone (preproANP) gene, *NPPA* (Gene ID: 4878), is located at the short arm of chromosome 1 (1p36.21) [4,5]. Human preproANP is a polypeptide with 151 amino acids. The first 25 amino acids of preproANP are a signal sequence, which are proteolytically removed during processing, and a 126-residue proANP peptide is then generated. The proANP peptide is the main storage form of ANPs in atrial granules. Specific pathophysiology signals, such as atrial wall mechanical stretching, and several hormones (angiotensin II, catecholamines, or vasopressin) can promote the release of proANP, which is rapidly cleaved by corin, leading to a COOH-terminal biologically active peptide with 28 amino acids, known as ANP [6] (Figure 1).

**Figure 2:**
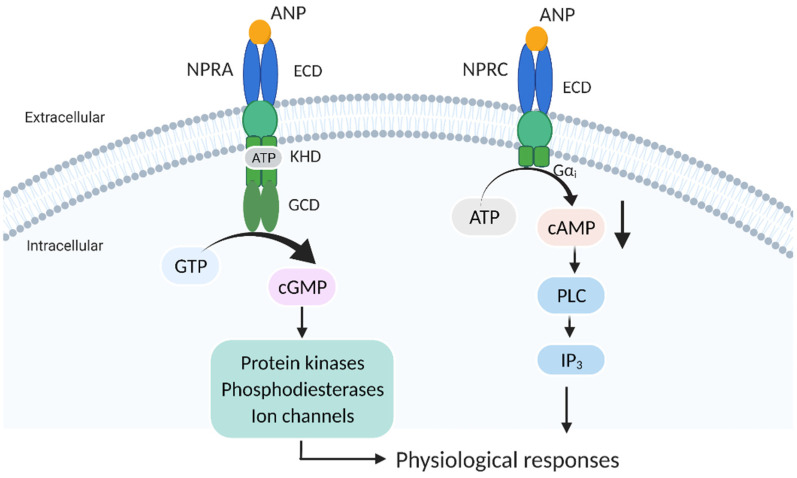
Diagram representing the downstream effects of ANPs and NPRs. ANPs bind to the extracellular ligand-binding domain (ECD) of NPRA, causing conformational change and phosphorylation of the intracellular protein-kinase homology domain (KHD), which then stimulates the guanylate cyclase catalytic domain (GCD) to convert GTP into cGMP, while the production of cGMP activates downstream effectors, such as protein kinases. ANPs bind to the ECD of NPRC to activate the inhibitory guanine nucleotide regulatory protein activator (Gαi) domain, thereby inhibiting adenylate cyclase activity and activating phospholipase C (PLC).

ANPs exert biological effects through interacting with two specific plasma membrane receptors: the principal receptor, or natriuretic peptide receptor A (NPRA), and natriuretic peptide receptor C (NPRC) [7]. NPRA is commonly expressed in a variety of tissues, including vasculature, heart, kidney, lungs, adrenal glands, adipose tissue, testis, liver, smooth muscle tissues, immune tissues, and some cancers [8,9]. The expression of NPRC is also ubiquitous, including the heart, lung, adrenal gland, brain, liver, and immune tissues [9,10]. NPRA consists of four functional domains: an extracellular ligand-binding domain (ECD), a single transmembrane-spanning region, an intracellular protein-kinase homology domain (KHD), and a guanylate cyclase catalytic domain (GCD) [11,12,13]. ANPs bind to the ECD, causing conformational change and the phosphorylation of six residues in the KHD, which then stimulates the GCD to convert GTP into intracellular second messenger cyclic guanosine phosphate (cGMP). Furthermore, the production of cGMP can activate downstream effectors, such as PKG, PDEs, and CGNs [14,15]. On the other hand, NPRC is a transmembrane receptor comprising a large ECD that has a similar structure as NPRA, a single membrane-spanning region, and a small intracellular portion of only 37 amino acids [16]. Previously, NPRC was regarded as functionally silent or a clearance receptor due to the lack of an intracellular guanylyl cyclase element. However, growing evidence indicates that there is an inhibitory guanine nucleotide regulatory protein activator (Gαi) domain residing in the intracellular portion of NPRC. The activation of the Gαi domain leads to the inhibition of adenylyl cyclase activity, as well as the activation of phospholipase C (PLC) [17,18] (Figure 2).

ANPs act as a key factor in the homeostatic control of blood pressure, electrolytes, and water balance by controlling the excretion of fluid and sodium [19]. They also coordinate with the renin–angiotensin–aldosterone system (RAAS) and the sympathetic nervous system [20,21,22]. In addition to the well-established hemodynamic functions, new biological effects of ANPs have emerged over the past two decades. ANPs play a vital role in modulating cell growth, counteracting oxidant-induced cell damage, as well as contributing to anti-inflammatory processes [23,24,25]. Moreover, growing findings have supported that ANPs show anti-tumor effects in various solid tumors, including prostate cancer, breast cancer, small-cell lung cancer, pancreatic cancer, et al. [26,27,28]. Studies have also demonstrated a significant correlation between the expression of NPRA and the growth of tumor cells [29]. Furthermore, several studies have reported that ANP treatment has a beneficial effect on alleviating severe side effects arising out of cancer therapy. Therefore, ANPs and their receptors have potential therapeutic value in tumors. This review focuses on the advances in ANP anti-inflammation and anti-cancer roles.

## 2. Immune-Modulatory Effects of ANPs

The ANP was first reported to be expressed in lymphoid organs in the late 1980s [30]. Since then, the expression of ANPs and their receptors have been identified in various immune organs and tissues, such as the thymus, spleen, tonsils, lymph nodes, bone marrow-derived macrophages, etc., providing convincing support for the interplay between ANPs and the immune system [5,14,24,31,32,33]. Given the fact that ANPs and their receptors are expressed in both innate and adaptive immune cells and are regulated by a variety of immunomodulatory factors, it is accepted that ANPs exert immune-modulatory effects in the tissue microenvironment by autocrine and paracrine mechanisms.

**Figure 3:**
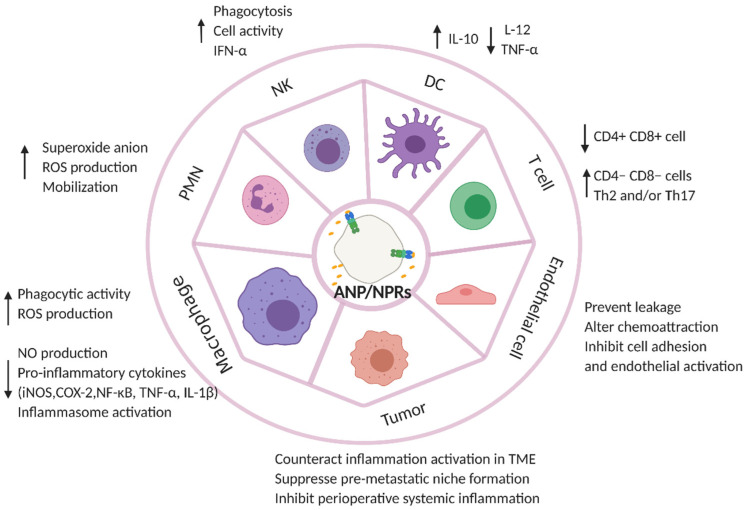
Immunomodulatory effects of ANPs. ANPs potentiate superoxide anion secretion, ROS production, and the mobilization of prime polymorphonuclear neutrophils (PMNs); ANPs tightly regulate the activity of macrophages, enhancing phagocytic activity and ROS production on the one hand, while reducing the production of NO and pro-inflammatory cytokines (iNOS, COX-2, TNFα, NF-κB, and IL-1β) and inhibiting inflammasome activation on the other hand. ANPs increase phagocytosis, cell activity, and the IFN-α release of nature killer (NK) cells; ANPs regulate maturation and differentiation of dendric cells (DCs) and promote anti-inflammatory cytokine (IL-10) release, but they inhibit pro-inflammatory cytokine (IL-12 and TNF-α) secretion. For adaptive immunity, ANPs decrease the percentage of CD4^+^CD8^+^ thymocytes and increase the CD4^−^CD8^−^ cell population. In addition, ANPs alter DC differentiation and subsequently polarize naive CD4^+^ cells toward the Th2 or Th17 phenotypes. ANPs protect endothelial barrier function, prevent leakage, alter chemoattraction and cell adhesion, and inhibit endothelial activation. ANPs counteract the occurrence of inflammation and inflammasome activation in the tumor microenvironment (TME), as well as inhibiting perioperative systemic inflammation and suppressing pre-metastatic niche formation.

The innate immune system serves as the front line of defense to detect and protect against invaders, such as viruses, bacteria, parasites, and toxins. It is comprised of barriers (epithelia), immune cells (i.e., monocytes, macrophages, neutrophils, granulocytes, dendritic cells, and nature killer cells), and anti-microbial agents (i.e., complement and opsonin). ANPs have been shown to protect endothelial barrier function, prevent leakage, alter chemoattraction and cell adhesion, and inhibit endothelial activation [34,35,36,37,38,39,40,41]. ANPs play an inhibitory role in the TNF-α-induced recruitment of pro-inflammatory cells. ANPs have been demonstrated to inhibit NF-κB activation to counteract the LPS- and TNF-α-induced expressions of adhesion molecules (i.e., E-selectin, intracellular adhesion molecules, and vascular cell adhesion molecules) in endothelial cells [42,43]. ANPs also inhibited p38MAPK activation to attenuate TNF-α-induced chemoattractant protein-1 (MCP-1) and IL-8 expression in endothelial cells [37]. In addition, ANP has inhibited the Rho inflammatory pathway or stimulated mitogen-activated protein kinase phosphatase-1 expression to prevent TNF-α- and LPS-induced endothelial permeability and cytoskeleton changes [34,40]. The fact that several immune cells produce ANPs and also express natriuretic peptide receptors suggested that ANPs may be a pleiotropic modulator of immune responses [44]. Several studies have reported that ANPs prime polymorphonuclear neutrophils (PMNs) to secrete superoxide anions by enhancing respiratory burst, potentiating ROS production and the mobilization of PMN induced by leukotriene B4 [23,24,45,46]. ANPs participate in immune response by tightly regulating the activity of macrophages. Mattana et al. showed that ANPs could modulate the phagocytic activity of macrophages by suppressing the uptake of IgG complexes at high concentration, whereas low concentrations of the ANP cloud increased the uptake of IgG complexes [47]. Moreover, ANPs have been shown to interact with NPRC, inducing sodium–proton exchanger isoform 1 (NHE-1) inactivation and decreasing intracellular pH (pHi) and subsequently eliciting a signaling pathway to active nicotinamide adenine dinucleotide phosphate (NADPH) oxidase and produce ROS [48,49,50]. ANPs have been shown to inhibit LPS-induced pro-inflammatory enzyme expression, such as nitric oxide synthase (iNOS) and cyclooxygenase-2 (COX-2). The inhibition of iNOS has been demonstrated to be mediated by inactivating NF-κB, an important transcription factor involved in the synthesis of iNOS, and increasing intracellular calcium levels to reduce iNOS mRNA stability, leading to the inhibition of NO. COX2 is inhibited via NPRC and has been associated with decreased cAMP production [47,51,52]. ANPs have significantly downregulated the expression of TNFα mRNA and have reduced the secretion of TNFα in macrophages by inhibiting the activation of both NF-κB and AP1 [53,54]. Mezzasoma et al. reported that the ANP/NPRA/cGMP axis downregulated LPS- and ATP-induced IL-1β secretion by inhibiting both NF-κB and NLRP3/caspase-1 activation in THP1 cells [55]. They further identified that the ANP/NPRA/cGMP/PKG-1 axis phosphorylated NLRP3 at Ser295 and broke down the inflammasome platform, leading to the counteraction of canonical inflammation activation. In addition, PDEs activated by ANP/NPRA/cGMP could interfere with the cleavage of Gasdermin D and Caspase-8, thereby disturbing non-canonical inflammation activation [56]. Furthermore, ANPs have been shown to augment the activity of natural killer (NK) cells in vitro and stimulate NK cells to secrete INF-α, which augments the killing of phagocytosed microbes by macrophages [57,58]. The ANP/NPRA axis also affects the immunomodulatory activity of dendritic cells (DCs). ANPs have decreased the production of IL-12 and TNF-α and enhanced the secretion of anti-inflammatory cytokine IL-10 in LPS-treated DCs, promoting DC differentiation into tolerogenic DCs and then polarizing naïve CD4^+^ T cells toward a Th2 phenotype [59,60,61]. As for their role in adaptive immunity, ANP treatment decreases the percentage of CD4^+^CD8^+^ thymocytes and increases the CD4^−^CD8^−^ cell population. In addition, ANPs have altered DC differentiation and subsequently polarized naïve CD4^+^ cells toward the Th2 or Th17 phenotypes [60,62] (Figure 3).

## 3. Anti-Cancer Effects of ANPs

A pilot report on the relationship between ANPs and cancer was first published in the late 1980s. Bradford et al. found that ANP expression was abundant in a patient with squamous cell carcinoma metastasis [63]. Since then, more and more researchers have begun to examine this subject. Rashed et al. reported that ANPs inhibited hepatoblastoma (HepG2) cell proliferation by upregulating NPRC and thereby reducing the intracellular concentration of cAMP [64]. In addition, Vesely et al. reported that ANPs reduced the number of cancer cells both in vitro and in vivo [65]. Moreover, KTH-222, a novel, small peptide derived from a motif in ANPs, exhibited a stronger effect than gemcitabine in inhibiting pancreatic cancer [66].

### 3.1. Modulation of Inflammation and Anti-Tumor Effects

Innate and adaptive immunity either promotes or inhibits various aspects of tumor development, as well as regulates responses to anti-cancer therapy [67]. The relationship between the immune system and carcinogenesis, as well as response to anti-tumor therapy, is intimate. The documenting of leukocytes within tumors, as observed in the 19th century by Rudolf Virchow, first linked cancer with inflammation [68]. This hypothesis gained clear evidence hereafter, and tumor-associated inflammation has been recognized as a hallmark of cancer [69]. Chronic inflammation drives tumorigenesis and promotes tumor progression and metastasis, as well as drug resistance [70].

ANPs can counteract the occurrence of inflammation and inflammasome activation in the TME to inhibit tumor development. Persistent inflammation is a potent risk factor for neoplastic transformation [71]. ANPs were shown to inhibit LPS-induced pro-inflammatory enzyme expression, NF-κB activation, and pro-inflammatory cytokine secretion [24]. Meanwhile, NPRA-disrupted mice have shown higher expressions of pro-inflammatory cytokines, such as TNF-α, IL-6, and TGF-β1 [72,73]. Subramanian et al. reported that 4 weeks of ANP treatment significantly inhibited skin cancer development in a mouse model of skin cancer. Significant reductions in the NF-κB activation levels, mast cell infiltration numbers, and MMP-2/9 levels in the skin tissues of mice treated with ANPs were observed, while the changes in serum lactate dehydrogenase-4, C-reactive protein, and enzymatic antioxidants (superoxide dismutase and catalase activities) were close to normal [74]. Chronic inflammation promotes prostate cancer progress and drug resistance [75]. Prostate cancer cells secrete extracellular vesicles (EVs) loaded with pro-inflammatory molecules to modify the tumor microenvironment and promote tumor progression [76]. Mezzasoma et al. reported that ANPs phosphorylated the NLRP3 receptor through the p38-MAPK pathway and then inhibited the inflammasome activation and IL-β maturation in PC3 cells, as well as reversing the inflammatory phenotypes of normal cells induced by EVs from PC3 cells [77].

ANPs are a potent agent to inhibit perioperative systemic inflammation and postoperative cancer recurrence. If the primary solid tumor meets surgical indications and the patient’s physical condition permits, the surgical removal of tumors remains a mainstay attempt to cure patients. However, surgical trauma not only provokes the detachment of cancer cells, leading to an increase in circulating tumor cell (CTC) count, but it also causes a severe systemic inflammatory reaction that speeds up the adhesion of CTCs to the endothelium of distant organs [78,79,80,81]. This is an important step in hematogenous metastases. Nojiri et al. reported that, in a large-scale observational clinical study, the perioperative administration of low-dose human ANPs reduced inflammatory responses and postoperative cardiopulmonary complications in patients receiving surgical treatment for lung cancer [82,83]. Interestingly, patients treated with ANPs had longer 2-year relapse-free survival times [84]. In the lungs of tumor-bearing mice, a significant inactivation of the ANP–NPRA pathway was noticed at the pre-metastatic niche, and ANP treatment downregulated pre-metastatic niche factors, thus preventing lung metastasis [84]. Therefore, the ANP–NPRA pathway may have potential therapeutic value in preventing the pre-metastatic niche formation of solid cancers.

NPRA signaling provides a critical link between inflammation and tumorigenesis. ANPs exert biological effects through interacting with two specific plasma membrane receptors: NPRA and NPRC. Kong et al. reported that the NH (2)-terminal peptide of the ANP prohormone NP73-102 exerted robust anti-inflammatory and anti-tumor effects by blocking the expression of NPRA [85]. In addition, in an endothelial-sprouting assay, an NPRA antagonist reduced NPRA expression and inhibited inflammation-induced angiogenesis by downregulating vascular endothelial growth factor (VEGF) and chemokine (C-X-C motif) receptor 4 (CXCR4). The accumulation of cancer-associated fibroblasts, endothelial cells, and macrophages decreased in an NPRA-disrupted mouse tumor microenvironment compared to WT mice, suggesting that NPRA signaling promoted tumor–stroma interaction. In addition, the absence of NPRA caused mesenchymal stem cells (MSCs) to fail to migrate to the TME. In contrast, significant increases in angiogenesis and tumorigenesis were noticed in NPRA-disrupted mice when co-implanting tumor cells with MSCs. These findings suggest that CXCR4 expression and stromal-derived factor 1α secretion are dependent on NPRA signaling [86]. Thus, NPRA signaling may be a potential therapeutic target for inflammation-associated tumorigenesis.

### 3.2. RAS-MEK1/2-ERK1/2 Kinase Cascade

Oncogenic mutated forms of Ras are detected in approximately 15% of cancers, and ERK hyperactivation can be seen in nearly one-third of human cancers, leading to deregulation of the RAS-mitogen-activated protein kinase (MEK)-extracellular-signal-regulated kinase (ERK) signaling pathway [87]. Sun et al. reported that ANP and LANP treatment could effectively inhibit the conversion of the RAS-GDP signal to the RAS-GTP signal in prostate cancer cells [88]. In addition, ANPs could suppress the activation of MEK1/2 and ERK1/2 in prostate cancer cells, and the inhibitory effect could be largely abolished by the cGMP antibody [89]. EGF and insulin were shown to stimulate ERK 1/2 kinases through mediating conversion of RAS-GDP to active RAS-GTP [90]. What is more, ANPs could block the activation of RAS and ERK 1/2 by mitogens such as insulin and epidermal growth factor (EGF) [91].

### 3.3. ANPs Interact with Other Transcription Factors and Cell Signaling Systems

#### 3.3.1. VEGF

Tumor cells and the surrounding stroma can secrete VEGF [92]. The overexpression of VEGF is related to tumor vascular density, invasiveness, metastasis, recurrence, and prognosis, and the blockading of VEGF may lead to a regression in the vascular network and the containment of tumor growth [93,94]. Given the fact that vascular endothelium cells express high levels of NPRA and that cell exposure to ANPs can counteract VEGF-induced endothelial cell proliferation signals, the ANP/NPR1 signal plays a critical role in the regulation of endothelial cell functional activity and proliferation [35]. Levin et al. showed that ANP inhibited the activation of several key signaling molecules, including ERK, JNK, and p38 members of the MAP kinase family, that were important for VEGF-induced angiogenesis [20]. ANPs could significantly block VEGF-induced endothelial cell proliferation and migration [95]. Nguyen et al. reported that ANPs could inhibit VEGF and VEGF receptor 2 in human cancer cell lines [96]. In addition, Mao et al. showed that ANPs combined with glipizide significantly suppressed breast cancer growth by inhibiting tumor-induced angiogenesis [97]. Moreover, Nakao et al. showed that high NPRA expression in tongue squamous cell carcinoma had a poorer prognosis, and NPRA was related to the expressions of VEGFA and VEGFC, which were associated with the invasion potential of tongue squamous cell carcinoma [98].

#### 3.3.2. Wnt/β-Catenin Signaling Cascade

The dysregulation of the Wnt/β-catenin signal caused by mutations or epigenetic changes contributes to the initiation and development of various human cancers [99]. ANPs downregulated β-catenin expression and caused a significant downregulation of c-Myc and cyclin D-1 transcriptions [65]. An acidic microenvironment has been identified as critical factor for the development of cancer. ANPs can modify the pH by inhibiting or stimulating NHE-1 [100,101]. Serafino et al. showed that ANPs induced NHE-1 inactivation, leading to an increase in intracellular acidity, inhibiting Wnt/β-catenin signaling through a frizzled-mediated activation that was on the upstream of the cascade, simultaneously [26].

#### 3.3.3. JNK and JAK/STATs Signaling

The c-JUN N-terminal kinase (JNK) pathway is involved in a range of pathological conditions, including inflammation and cancer progression [102]. ANPs could reduce the expression of JNK2 in human prostate adenocarcinoma and small-cell lung cancer cells [28]. The signal transducer and activator of transcription (STAT3) signal is constitutively activated during cancer development and is associated with different hallmarks of cancer [103]. Lane et al. showed that ANPs inhibited the expression of STAT3 in human cancer cells [65].

#### 3.3.4. ROS Production

Cancer cells show higher level of ROS than normal cells, partly due to high metabolic activity, peroxisomal activity, and mitochondrial dysfunction, which contributes to tumorigenesis, tumor progression, and metastasis, as well as drug resistance [104,105,106]. When the levels of ROS fluctuate, cancer cells more easily reach their oxidative stress thresholds compare to normal cells, resulting in oxidative-stress-induced cell death [107,108]. In hepG2 cells, increased expression of ANP-inhibited cell growth was seen through the upregulation of NPRC [64]. Baldini et al. showed that, in ANP-treated HepG2 cells, a significant decrease in pHi, enhancement in the PLD activity, and an increase in the ROS level were observed simultaneously [109]. Furthermore, ANP-induced ROS generation via the involvement of the NADPH oxidase system subsequently inhibited the caspase-3 enzyme and switched HepG2 cell death from apoptosis to necrosis [110]. However, Kiemers et al. showed that ANP had a cytoprotective effect on liver cells after ischemia reperfusion injury [111], which might be attributed to the differences between tumor cells and normal cells in terms of threshold signal, NPR-C expression, and intracellular ROS level [23].

#### 3.3.5. KCNQ1 Expression

Plasma K^+^ channels are important in tumor cell proliferation [112,113]. Zhang et al. showed that ANPs played a pleiotropic role in gastric cancer cell proliferation, and the physiological concentration of ANPs upregulated the expression of the potassium-voltage-gated channel and KQT-like subfamily member 1 (KCNQ1) to promote AGS cell proliferation, while the pharmacological concentration of ANP significantly downregulated KCNQ1 expression to inhibit AGS proliferation [114]. Furthermore, the knockdown of NPRA decreased the protein level of KCNQ1 and the voltage-gated outward K^+^ current [115].

#### 3.3.6. MMP9 Expression

**Figure 4:**
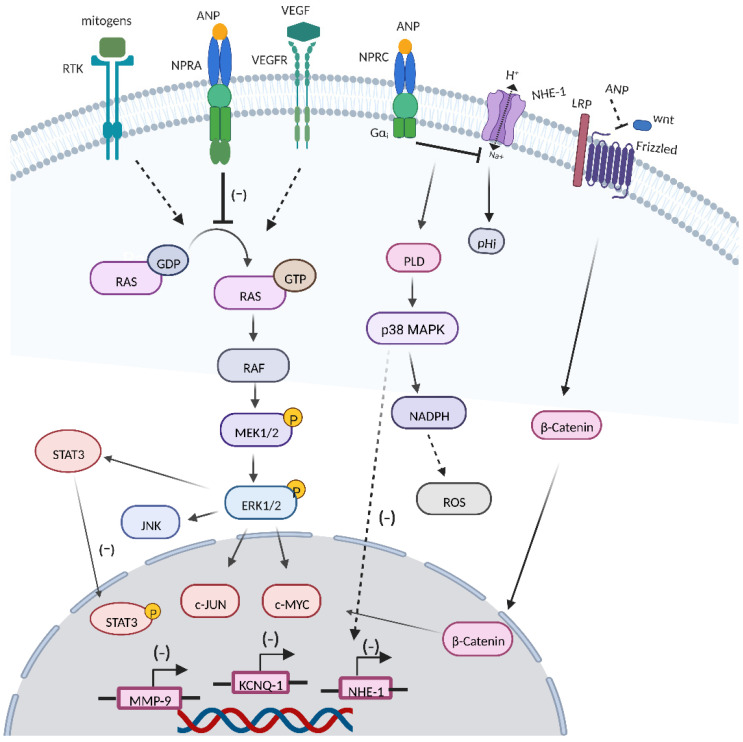
Roles of ANPs in cancer. ANPs exert antineoplastic potential by inhibiting the conversion of GDP-RAS to GTP-RAS, the RAS-MEK1/2-ERK1/2 kinase cascade, and cross-talk between the RAS-MEK1/2-ERK1/2 kinase cascade and VEGF, β-catenin, JNK, WNT, and STAT3. In addition, ANPs also modulate inflammation, ROS production, KCNQ1, and MMP9 expression.

The dysregulation of MMP9 is related to several hallmarks of cancer, including inflammation, angiogenesis, tumor growth, and metastasis [116]. Li et al. reported that ANPs significantly inhibited gastric cancer cell migration and invasion by downregulating the hedgehog-signaling-mediated activation of MMP9 [117]. In vivo, ANP treatment significantly reduced NF-κB activation levels, mast cell infiltration numbers, and MMP2/9 levels in the skin tissues of a skin cancer mouse model [74] (Figure 4).

## 4. ANPs Attenuate Side Effects of Cancer Therapy

It has been reported that ANPs exert protective effects in a variety of organs, including the heart, lungs, kidneys, and blood vessels [118,119,120,121,122,123,124]. Several clinical studies have shown the prophylactic effects of ANP therapy on contrast-induced nephropathy, renal function, postoperative cardio-renal events following cardiac surgery, postoperative cardiopulmonary complications, and cancer recurrence after curative lung cancer surgery [125,126]. Acute kidney injury and myelosuppression are the major toxicities of cisplatin therapy. Nojiri et al. showed that ANP treatment inhibited the increase in serum urea nitrogen/creatinine and urinary albumin/creatinine, as well as downregulating IL-1β, IL-6, and MCP-1 in renal tissue, thus alleviating renal dysfunction and renal tubular necrosis induced by cisplatin [127]. ANPs also significantly reduced the decrease in white blood cell count in the peripheral blood, as well as the decrease in CFU-GM colonies in the bone marrow, after cisplatin injection [128]. The detailed mechanism of ANPs for attenuating cisplatin toxicity needs further study.

Cytokine release syndrome (CRS), an acute systemic inflammatory response, is a lethal complication of several immunotherapies [129,130,131,132]. CRS is an obstacle to the clinical application of certain tumor biotherapies. Staedtke et al. showed that catecholamines orchestrated immune disorders caused by oncolytic bacteria and LPS through a self-amplifying loop in macrophages. ANP treatment abolished catecholamines’ self-amplifying loop, reduced the secretion of inflammatory cytokines, and counteracted the markedly enhanced inflammatory response caused by LPS in combination with adrenaline. Therefore, ANPs protected mice from the life-threatening toxicity of CRS but did not impair the therapeutic response [133]. These data indicate that ANPs exert an anti-inflammatory effect produced by biotherapeutic agents by inhibiting macrophage activation. The fact that ANPs can effectively attenuate CRS may provide hope for the clinical utility of biological therapies that are limited by CRS.

## 5. Future Perspectives

The current review depicted the pleiotropic role of ANPs in immune modulation and neoplastic prevention. However, some questions regarding the pleiotropic roles of ANPs in immune responses and neoplastic prevention should be elucidated. Firstly, the detailed role of ANPs in exerting anti-inflammatory and immune-modulatory effects in the tumor microenvironment should be elucidated. Secondly, the more detailed mechanism by which ANPs suppress cancer metastasis needs to be dissected. Thirdly, the downstream effects of ANP–NPRA signaling on antineoplastic factors need to be dissected and provided with more evidence. Since ANPs have a robust effect on inflammation inhibition, the pro-inflammation effect of the NPA receptor, NPRA, reported in some studies needs more verification. It will be interesting to reveal the underlying mechanisms of the opposite roles of NPA and NPRA in inflammation.

## 6. Conclusions

The relationship between the immune system and carcinogenesis, as well as response to anti-tumor therapy, is intimate. Through autocrine and paracrine mechanisms, ANPs inhibit the synthesis and secretion of proinflammatory cytokines, as well as the expression of several adhesion molecules, thus exhibiting a fascinating ability to prevent inflammation-related tumorigenesis and cancer recurrence. This can stimulate research into the design of anticancer agents based on ANP/NPRA signaling, as the relationship is intimate between various cancers and inflammation. By examining the anti-tumor potential of ANPs, we provided sufficient information that ANPs inhibit the conversion of GDP-RAS to GTP-RAS, the RAS-MEK1/2-ERK1/2 kinase cascade, and the RAS-MEK1/2-ERK1/2 kinase cascade cross-talk with VEGF, β-catenin, JNK, WNT, and STAT3. In addition, ANPs also modulate inflammation, ROS production, and KCNQ1 and MMP9 expression. ANPs have anti-proliferative and anti-inflammatory properties without severe adverse effects, which makes ANPs a potential target for the treatment of malignant tumors because many current chemotherapy drugs have large cytotoxicity, side effects, and too high a cost in life quality with limited prognostic improvement.

## Data Availability

Not applicable.
